# Viral Hepatitis Strategic Information to Achieve Elimination by 2030: Key Elements for HIV Program Managers

**DOI:** 10.2196/publichealth.7370

**Published:** 2017-12-15

**Authors:** Yvan Hutin, Daniel Low-Beer, Isabel Bergeri, Sarah Hess, Jesus Maria Garcia-Calleja, Chika Hayashi, Antons Mozalevskis, Annemarie Rinder Stengaard, Keith Sabin, Hande Harmanci, Marc Bulterys

**Affiliations:** ^1^ HIV and Hepatitis Department World Health Organization Geneva Switzerland; ^2^ Regional Office for Europe World Health Organization Copenhagen Denmark; ^3^ Joint United Nations Programme on HIV/AIDS Geneva Switzerland

**Keywords:** hepatitis, HIV, surveillance, evaluation

## Abstract

Evidence documenting the global burden of disease from viral hepatitis was essential for the World Health Assembly to endorse the first Global Health Sector Strategy (GHSS) on viral hepatitis in May 2016. The GHSS on viral hepatitis proposes to eliminate viral hepatitis as a public health threat by 2030. The GHSS on viral hepatitis is in line with targets for HIV infection and tuberculosis as part of the Sustainable Development Goals. As coordination between hepatitis and HIV programs aims to optimize the use of resources, guidance is also needed to align the strategic information components of the 2 programs. The World Health Organization monitoring and evaluation framework for viral hepatitis B and C follows an approach similar to the one of HIV, including components on the following: (1) context (prevalence of infection), (2) input, (3) output and outcome, including the cascade of prevention and treatment, and (4) impact (incidence and mortality). Data systems that are needed to inform this framework include (1) surveillance for acute hepatitis, chronic infections, and sequelae and (2) program data documenting prevention and treatment, which for the latter includes a database of patients. Overall, the commonalities between HIV and hepatitis at the strategic, policy, technical, and implementation levels justify coordination, strategic linkage, or integration, depending on the type of HIV and viral hepatitis epidemics. Strategic information is a critical area of this alignment under the principle of what gets measured gets done. It is facilitated because the monitoring and evaluation frameworks for HIV and viral hepatitis were constructed using a similar approach. However, for areas where elimination of viral hepatitis requires data that cannot be collected through the HIV program, collaborations are needed with immunization, communicable disease control, tuberculosis, and hepatology centers to ensure collection of information for the remaining indicators.

## Introduction

The Global Hepatitis Report [1] indicated that in 2015, 1.34 million persons died from the consequences of viral hepatitis. Hence, mortality from viral hepatitis is at par with tuberculosis, and higher than the HIV infection or malaria [2]. More than 90% of this burden is due to cirrhosis and hepatocellular carcinoma, the sequelae of infections with hepatitis B virus (HBV) and hepatitis C virus (HCV) [1]. In May 2016, the World Health Assembly endorsed the Global Health Sector Strategy (GHSS) for 2016-2021 on viral hepatitis that proposes to eliminate viral hepatitis as a public health threat by 2030 (elimination is defined as a 90% reduction in new chronic infections and a 65% reduction in mortality compared with the 2015 baseline.) [3]. The World Health Organization (WHO) conceived this strategy using the same universal health coverage and public health frameworks that were used for strategies for HIV [4] and sexually transmitted infections (STIs) [5]. These 3 strategies were developed and adopted together; their 5 strategic directions are as follows: (1) information for focused action, (2) interventions for impact, (3) delivering for equity, (4) financing for sustainability, and (5) innovation for acceleration.

To eliminate viral hepatitis as a public health threat, the GHSS proposes to scale up prevention interventions currently available, such as universal immunization of children against hepatitis B, including a timely birth dose to prevent mother-to-child transmission [6]. In addition, it also introduces newer programmatic components, such as testing and treatment (older policy responses did not address chronic infection). Testing and treatment services for HBV, HCV, and HIV infections can use similar programmatic and delivery approaches. Thus, implementation of hepatitis and HIV interventions at the global, regional, and country level is being progressively considered as a coordinated approach where it is possible and improves impact and efficiency.

Strategic information can be defined as data collected at all administrative levels to inform policy and program decisions. The WHO published consolidated strategic information guidelines for HIV, including a monitoring and evaluation framework that includes 10 core indicators [7]. In 2016, the WHO also published guidance for strategic information in the field of viral hepatitis, including surveillance [8], and monitoring and evaluation [9]. This paper summarizes the approach proposed by the WHO to collect, analyze, and use viral hepatitis strategic information so that HIV and hepatitis services have aligned approaches in terms of service delivery and data management.

### HIV, HBV, and HCV: Similarities and Differences

#### Similarities

In infected persons, HIV, HBV, and HCV are present in blood and most body fluids. Hence, these viruses share a number of modes of transmission, including mother-to-child, blood-borne, and sexual. HIV, HBV, and HCV lead to infections that may be silent for a number of years before consequences of infections lead to fatal sequelae. HIV, HBV, and HCV infections can be prevented with safer sex, interventions to reduce mother-to-child transmission, blood safety, standard universal precautions in health care and other settings, and harm reduction interventions for people who inject drugs (PWID). Specific risk groups (eg, PWID and men who have sex with men) are often disproportionally affected by HIV, HBV, and HCV and can be reached through coordinated health sector and community-based services, using the people-centered approaches recommended by the Sustainable Development Goals (SDGs). Mortality from chronic HBV and HCV infections can be reduced by testing and treatment, similar to HIV.

#### Differences

Aside from the many similarities between HIV, HBV, and HCV, there are a number of differences that need to be understood for optimized program implementation, including surveillance, monitoring, and evaluation.

##### Burden

Globally, HBV and HCV infections are more prevalent than HIV infection (Table 1) and may be distributed differently by region. However, in the absence of treatment, the case fatality of HIV is higher, and deaths tend to occur earlier in the course of the infection (50% within 10 years of infection), and therefore at an earlier age [2]. Regional variations in the differences in prevalence and mortality between HIV, HBV, and HCV have implications in the way that these areas of work may relate, from coordination to strategic linkages or integration, according to the magnitude of the epidemics [10].

##### New Infections

New infections with HIV are asymptomatic in most cases or may result in a nonspecific illness. Among adults, new infections with HBV and HCV lead to acute hepatitis in less than 50% of instances. The burden of disease from acute hepatitis is small in comparison with the burden from chronic infections [1]. However, if identified and reported in the context of enhanced case reporting, these cases of acute hepatitis can provide critical information on incidence trends and risk factors [8]. For HBV, this is facilitated by the availability of a marker of recent infection (IgM antibodies against HBV core antigen [anti-HBc IgM]). For HCV, in the absence of a biomarker of recent infection, case definitions are available for the surveillance of acute hepatitis C. The case definition of acute hepatitis C is an acute hepatitis that is non-A, non-B, and non-E and positive for HCV biomarkers [8].

##### Chronic Infections

In the case of HIV infection, most untreated persons will develop acquired immunodeficiency syndrome (AIDS) (with the exception of about 5% who are “elite controllers”) [11]. In the case of hepatitis, some patients newly infected with HBV and HCV spontaneously clear infection (80%-95% for HBV and about 20% for HCV) [8]. Hence, for hepatitis, it is relevant to use biomarkers to differentiate evidence of past or present infection from evidence of present (current) infection. Markers of past or present infection include antibody to HBV core antigen (total anti-HBc) and antibody to HCV (anti-HCV). Markers of present infection include hepatitis B surface antigen (HBsAg) and HCV RNA. As for HIV, the age at which HBV and HCV infection occurs influences the natural history. For HBV, infections acquired in the first 5 years of life most often lead to chronic infections and sequelae [12]. They account for the largest proportion of the burden of disease affecting adults. In contrast, the incidence of HCV infection in children is generally low, and infections acquired in childhood are probably of a better prognosis [13].

**Table 1:** Key characteristics of hepatitis B virus (HBV), hepatitis C virus (HCV), and HIV infection, including epidemiology, clinical manifestations, biomarkers, routes of transmission, prevention, and treatment.

Characteristics		Hepatitis B virus (HBV)	Hepatitis C virus (HCV)	HIV
**Estimates of epidemiology and burden**				
	Prevalence (millions of infections)	257	71	33
	Annual mortality (millions of deaths)	0.887	0.399	1.341
**Clinical manifestations**				
	Clinical manifestations of new infections	Acute hepatitis (uncommon in <5 years, 50% of new infections among persons aged ≥5 years)	Acute hepatitis (<20% of new infections)	Nonspecific clinical manifestations of acute HIV infection
	Spontaneous clearance of infection	80%-95% of new infections	20% of new infections	None
	Long-term complications	Cirrhosis and hepatocellular carcinoma	Cirrhosis and hepatocellular carcinoma	Chronic infection leading to immune suppression (AIDS^a^)
**Biomarkers**				
	New/recent infection	IgM anti-HBc^b^	None^c^	Some options available with nucleic acid testing or “recency” serological tests
	Past or present infection	Total anti-HBc	Anti-HCV	Anti-HIV
	Present infection	HBsAg^d^	HCV RNA^e^ or HCV core antigen	Anti-HIV
**Routes of transmission**				
	Perinatal	Delivery and uncommonly, before birth	Uncommon^f^	Before, during, and after birth
	Sexual	++^g^	+/−^g^ Common in HIV-infected men who have sex with men	+++
	Blood-borne	++++	+++	++
	Vaccine	Yes	No	No
**Approach to prevention**				
	Mother-to-child transmission	Universal immunization of infants, starting at birth +/− HBIg^h^ +/− antivirals during pregnancy	Cure mothers before pregnancy	Test and treat
	Prevention of other new infections	Universal immunization, safe injection practices, infection control, blood safety, and safe sex	Safe injection practices, infection control, blood safety, and safe sex	Safe sex, voluntary surgical male circumcision, safe injection practices, infection control, blood safety, preexposure prophylaxis
Treatment		Lifelong treatment with nucleos(t)ides analogues	Treatment available leading to cure after short course	Lifelong treatment with a combination of medicines

^a^AIDS: acquired immunodeficiency syndrome.

^b^Anti-HBc: antibody to the hepatitis B core antigen.

^c^RNA or core antigen positive in the absence of anti-HCV suggests recent HCV infection.

^d^HBsAg: hepatitis B surface antigen.

^e^RNA: ribonucleic acid.

^f^Risk of mother-to-child transmission is higher among HIV-infected pregnant women.

^g^symbol +/- and + quantifies the importance of transmission.

^h^HBIg: Hepatitis B immune globulin.

##### Sequelae

Ten years after initial infection, without treatment, about 50% of persons with HIV infection will have developed AIDS [14]. AIDS may be recognized by infectious disease physicians or the diagnosis may be missed if the patient dies of other opportunistic infections. In contrast, HBV and HCV infections lead to cirrhosis and hepatocellular carcinoma, two conditions that may be primarily managed by internists or gastroenterologists rather than infectious disease physicians in settings where patients have access to specialized care. The duration between infection and death is longer in the case of HIV, in the range of 20 to 30 years [15-17]. Other cofactors (eg, alcohol use, metabolic syndrome, toxins, and substance abuse) can also affect the risk of developing cirrhosis or hepatocellular carcinoma. As a result, the hepatitis virus infection that led to these sequelae may not be recognized or reported as the cause of death. This complicates the measurement of the burden of disease associated with HBV and HCV infection [18]. Historically, the Global Burden of Disease took a number of years to attribute cirrhosis and hepatocellular carcinoma to HBV and HCV infections [18], and the current approach still does not take into account the extrahepatitic manifestation of the HCV infection [19].

##### Transmission and Prevention

HIV, HBV, and HCV can be transmitted through the same routes (Table 1). However, the relative importance of these routes varies according to the viruses. HBV is often transmitted through percutaneous, sexual, and perinatal routes. HIV is most often transmitted through sexual contacts. Injection drug use is also a common route of HIV transmission, but health care injections are an uncommon source of infection, even though outbreaks have occurred [20,21]. HCV is more often transmitted through the percutaneous route and accounts for the largest number of health care injections–associated infections [20]. Despite these differences in the relative importance of the various routes of transmission, methods of primary prevention do not differ (Table 1). However, for HBV, a safe and effective vaccine is also available [6]. Three doses of hepatitis B vaccine, with the first one administered soon after birth, can prevent the majority of chronic HBV infections [6]. Recommendations for the prevention of mother-to-child transmission also vary for the 3 viruses: immunoprophylaxis to prevent mother-to-child transmission of HBV and antiretroviral treatment for mother and child against HIV. Mother-to-child transmission of HCV is rare, and there is no specific approach to prevent it [22].

##### Treatment

As of 2016, treatment for HBV infection was in most cases lifelong. A single antinucleos(t)ide with a high barrier to resistance is sufficient to achieve viral suppression for patients who are eligible for treatment [23]. Hence, from a data management point of view, the approach to monitoring treatment is similar to HIV. Since 2013, HCV can be cured through a short-course treatment of a few months [13]. This revolution in curative treatment led to a new momentum for elimination. Hence, the approach to monitoring HCV treatment is more similar to curable infections such as tuberculosis. All HCV-infected patients are potentially eligible for treatment. Coinfections with any combination of the 3 viruses require specific management considerations (see Table 1).

### GHSS on Viral Hepatitis

The 2014 World Health Assembly requested the WHO to examine the feasibility of eliminating hepatitis B and C. In 2015, the SDGs committed to combating viral hepatitis (Target 3.3) [24]. As a result, the WHO coordinated a think tank to examine options. As part of this work, a mathematical model suggested that if the viral hepatitis response reached 5 synergistic prevention and treatment service coverage targets (Table 2), hepatitis B and C could be eliminated as a public health threat [25,26]. These 5 interventions now endorsed by a World Health Assembly resolution are as follows: (1) hepatitis B immunization, (2) prevention of mother-to-child transmission of HBV, (3) blood and injection safety, (4) prevention of transmission among persons who inject drugs through comprehensive harm reduction services, and (5) testing and treatment. Service coverage targets for 2030 with respect to testing and treatment are 90% of patients diagnosed and 80% of patients eligible treated (using a logic similar to 90% of people with HIV diagnosed, 90% of people diagnosed with HIV treated, and 90% of those on treatment virally suppressed by 2020 for HIV targets) [4].

### Monitoring Framework for Viral Hepatitis B and C

#### Levels and Indicators

The WHO proposed a monitoring and evaluation framework for viral hepatitis B and C (Multimedia Appendix 1) that, similar to the 10 core indicators recommended for HIV, follows the result chain, from (1) context and needs to (2) input, (3) output and outcomes, and (4) impact [9].

##### Context

Given the focus on chronic HBV and HCV infections, prevalence of infection in the population is the best reflection of context and needs (C.1), similar to what is being used for HIV.

##### Input

Input refers to systems and resources available for hepatitis elimination. One of the most challenging service coverage targets of the GHSS is to increase the proportion diagnosed among those who are infected (30% by 2020 and 90% by 2030; Table 2). This will require a substantial increase in the capacity to test individuals for HBV or HCV infection. Hence, the WHO selected the number of health care facilities that are able to test for HBV and HCV infections per 100,000 people as a core input indicator (C.2).

##### Output and Outcomes

###### Prevention

Three core prevention indicators reflect modes of transmission that are key for hepatitis. These include coverage of the third dose of hepatitis B vaccine and of timely birth dose (hepatitis B vaccine birth dose or other methods to prevent mother-to-child transmission of HBV infection) (C.3), the number of syringe and needle sets distributed to persons who inject drugs (C.4) [27], and the proportion of safe injections at the health care facility level (C.5) [28]. Indicators reflecting sexual transmission are not included among these core indicators because sexual transmission accounts for a lower proportion of HBV and HCV infections than HIV [29].

**Table 2:** Global service coverage targets that would eliminate HBV^a^ and HCV^b^ as public health threats, 2015-2030.

Target areas				Baseline 2015	2020 target	2030 target
**Service coverage**						
	**Prevention**					
		Three-dose HBV for infants (coverage %)		84	90	90
		Prevention of mother-to-child transmission of HBV: hepatitis B birth-dose vaccination or other approaches (coverage %)		39	50	90
		**Blood and injection safety**				
			Blood safety: donations screened with quality assurance (coverage %)	97	95	100
			Injection safety: use of engineered devices^c^ (coverage %)	5	50	90
		Harm reduction (sterile syringe/needle set distributed per person per year for people who inject drugs [PWID])		20	200	300
	**Treatment**					
		Diagnosis of HBV and HCV (coverage %)		9-20	30	90
		Treatment of HBV and HCV		7%-8%	5 million (HBV) and 3 million (HCV)	80% eligible treated
**Impact leading to elimination**						
	Incidence of chronic HBV and HCV infections			6-10 million	30% reduction	90% reduction
	Mortality from chronic HBV and HCV infections			1.34 million	10% reduction	65% reduction

^a^HBV: hepatitis B virus.

^b^HCV: hepatitis C virus.

^c^Although the service coverage target is about output (adoption of reuse prevention injection devices), the C.5 indicator focuses on outcome (provision of safe injections).

###### Cascade of Care

Using an approach very similar to HIV, the indicators of the cascade of care (for HBV) or cure (for HCV) include the proportion of people living with viral hepatitis diagnosed (C.6), treatment coverage (in the case of HBV infection, C.7.a) or initiation (in the case of HCV infection, C.7.b), and treatment outcome, which includes the proportion of people on treatment who are virally suppressed (C.8.a, for HBV infection) or the proportion of people cured among those who completed treatment (C.8.b, for HCV infection). In the case of HBV infection, a high proportion of people with chronic infection are not eligible for treatment [23]. Hence, the coverage of linkage to care among those diagnosed is also important as some diagnosed patients will require long-term follow-up to determine when they become eligible for treatment (Indicator A.8) [9].

##### Impact

Similar to HIV, the results chain is linked to impact (incidence and mortality). These are the parameters upon which elimination is defined (ie, 90% reduction in incidence and 65% reduction in mortality compared with the 2015 baseline). The WHO designated the cumulative incidence of HBV infection among children at 5 years of age (C.9.a) to evaluate progress in “combatting hepatitis” as per the SDG [30]. The restriction to this age group is because HBV infections among children below 5 years of age contribute most to the burden of chronic infections among adults [12]. The incidence of HCV infection is measured in the whole population (C.9.b). There is a mortality indicator designated for HBV (C.10.a) and HCV (C.10.b).

#### Practical Implementation of the Monitoring and Evaluation Framework

The data systems needed to report against the core indicators of the monitoring and evaluation framework for viral hepatitis (Table 2) include the following:

- C.1: regular biomarker surveys to estimate the prevalence of HBV and HCV infection (see the Surveillance for Chronic Infections section below). This could be coordinated with HIV surveys [31] and surveys to evaluate the impact of hepatitis B immunization [32].
- C.2: program data or health care facility surveys to estimate the ratio of facilities that can test for HBV and HCV infection per 100,000 population. This is compatible with the indicators for laboratory diagnosis capacity measured in the context of the SDGs and compatible with the WHO-recommended approach for survey of health care facilities (Service Availability and Readiness Assessment [SARA]) [33].
- C.3: routine data from the Expanded Program on Immunization to estimate vaccine coverage (estimates generated by WHO and UNICEF [United Nations International Children’s Emergency Fund] are available on the Web) [34].
- C.4: program data on needle and syringe distribution that reflects broader harm reduction activities (using the same data sources as for HIV).
- C.5: population surveys or health care facility surveys to estimate the proportion of safe injections [28]. This can be coordinated with other population surveys or with SARA [33].
- C.6-7-8: data from a patient’s database to monitor the cascade of diagnosis and treatment. In the absence of a separate database, unique identifiers allow patients to be tracked along the cascade and link patients across disease registries, clinics, and vital statistics. This can be coordinated with monitoring of patients with HIV infection where clinics routinely diagnose and treat hepatitis and HIV or in epidemics characterized by high prevalence of coinfection [7].
- C.9.a: biomarker survey in children who were vaccinated to estimate the impact of hepatitis B immunization on the cumulated incidence of chronic HBV infection [32]. This may be difficult to coordinate with household surveys that incorporate HIV testing (AIDS indicator surveys or public health impact assessments) because of sample size requirements and age group considerations [32].
- C.9.b: modeling estimates from biomarker surveys in the population (or specific groups) and trends on enhanced case reporting for acute hepatitis C8 to estimate the incidence of HCV infection (see the Surveillance for Acute Hepatitis section below). This could be coordinated with HIV modeling activities, where relevant, depending on the type of epidemic [31].
- C.10: combination of vital statistics data on the mortality from hepatocellular carcinoma and cirrhosis processed with data on the prevalence of HBV and HCV infection in patients with these sequelae (See the Surveillance for Sequelae section below).

### Surveillance for Viral Hepatitis

Surveillance for viral hepatitis refers to the systematic, ongoing collection, transmission, analysis, and use of epidemiological data on viral hepatitis [8]. Surveillance focuses on epidemiological parameters such as incidence, prevalence, and mortality. The WHO monitoring and evaluation framework for viral hepatitis B and C addresses other components, such as behaviors. Surveillance for viral hepatitis has 3 components (see Table 3) that correspond to the natural history of HBV and HCV infections. These are as follows: (1) surveillance for acute hepatitis that reflects new infections, (2) surveillance for chronic, prevalent infections, and (3) surveillance for sequelae, including cirrhosis and hepatocellular carcinoma. These 3 components contribute to a comprehensive picture of the epidemiological situation of viral hepatitis. They feed the viral hepatitis monitoring and evaluation framework with key data (Multimedia Appendix 1). However, they may be implemented by different actors in a country’s public health system. Thus, these different actors must coordinate to consolidate and triangulate pieces of information from different sources. As for all surveillance activities, standardized case definitions are essential to viral hepatitis surveillance, including surveillance for acute hepatitis and chronic infections (Table 4).

#### Surveillance for Acute Hepatitis

The majority of new infections with hepatitis viruses are asymptomatic or undiagnosed. However, surveillance for acute hepatitis can be informative through capturing a constant fraction of cases. Surveillance for acute hepatitis is usually implemented in the context of communicable disease surveillance systems. It differs from reporting of newly diagnosed cases of chronic infections that must be handled through patient registries (see the Patients Database section below). The WHO formulated standardized case definitions for surveillance for acute hepatitis (Table 4) [8]. In the field of surveillance for acute hepatitis, 2 different activities need to be distinguished: syndromic surveillance and enhanced case reporting.

*Syndromic surveillance* for undifferentiated acute viral hepatitis involves reporting by all health care facilities of clinical cases of acute hepatitis in the absence of in vitro diagnosis. This type of surveillance may detect large outbreaks, which are usually outbreaks of hepatitis A or E. However, surveillance for undifferentiated acute viral hepatitis is not essential to eliminate hepatitis B and C as public health threats.

*Enhanced case reporting* involves reporting by health care facilities of cases of acute hepatitis, by type (ie, A, B, C, D, or E), with in vitro diagnosis (ie, IgM tests) and collection of information of possible exposures. Cases of acute hepatitis are uniquely informative as they denote recent infections. Hence, collection of information on possible exposures during the referent exposure period (or the incubation period) informs on sources of infection. Enhanced case reporting may be difficult to implement countrywide. Hence, countrywide enhanced case reporting is mostly limited to high-income countries. However, in resource-limited settings, it can be done in sentinel sites where there is access to good in vitro diagnosis (eg, emergency departments). Enhanced case reporting allows description of trends in type-specific acute hepatitis and contributes to the generation of hypotheses regarding prevailing risk factors in a given setting. If enhanced case reporting is in place countrywide, it is probably implemented in the context of the communicable disease surveillance system. In the absence of a national system, a small number of sentinel sites may be needed, for example, in selected hospitals where IgM in vitro diagnosis is available for the diagnosis of recent infections. Monitoring the strength of the association between health care or injection drug use and new HBV or HCV infection can be beneficial to HIV prevention. HBV [35] and HCV [36] are more easily transmitted through percutaneous exposures than HIV [37]. New HBV and HCV infections are also easier to detect. Therefore, documentation of transmission of HBV or HCV through these routes using surveillance of acute hepatitis can provide early warning signals for the risk of HIV transmission, as recently illustrated with the outbreak of HBV and HCV associated with injection drug use in the United States [38,39].

**Table 3:** Surveillance activities needed to describe the epidemiology of viral hepatitis, including hepatitis B and hepatitis C.

Parameter	Activities that contribute to surveillance for viral hepatitis				
	Surveillance for acute hepatitis that reflect new infections		Surveillance for chronic, prevalent hepatitis		Surveillance for sequelae
Activities	Syndromic surveillance in the general population; Event-based surveillance^a^	Enhanced case reporting (with in vitro diagnosis and collection of information on risk factors) countrywide or in sentinel sites^b ^	Case reporting from laboratories or health care facilities	Regular biomarker surveys	Combination of data from cancer registries, death certificates, and testing of cirrhosis and HCC^c^ patients for HBV^d^ and HCV^e^ infection
Population under surveillance	Persons presenting with acute hepatitis in health care facilities (discrete onset of symptoms)	Persons presenting with acute hepatitis in health care facilities (discrete onset of symptoms)	Persons without acute symptoms tested in health care facilities/laboratories	Person without acute symptoms tested during population surveys	Persons diagnosed with cirrhosis and HCC
Usual implementer	Communicable disease surveillance	Communicable disease surveillance (if countrywide); hepatitis program (if sentinel sites)	Communicable disease surveillance and/or hepatitis program	Hepatitis program in coordination with the other actors implementing biomarker surveys	Hepatitis program collating data from various different sources, including vital registration
Case definitions to use (see Table 4)	Presumptive case of acute hepatitis	Confirmed case of acute hepatitis (by type)	Chronic HBV and HCV infection; serological evidence of past or present HCV infection	Chronic HBV and HCV infection; serological evidence of past or present HCV infection	Cases of HCC or cirrhosis with chronic HBV or HCV infection
Objective of the surveillance activity	Detect outbreaks	Describe trends in type-specific acute hepatitis^f^ and identify risk factors	Estimate the proportion of chronically infected persons who have been identified	Estimate the burden of chronic infections; model incidence trends	Estimate the incidence of HCC and cirrhosis

^a^In vitro diagnosis needs to be organized on a sample of cases when an outbreak is reported.

^b^High-quality data (ie, reliable in vitro diagnosis and good information on risk factors) from a smaller number of tertiary centers is preferable and more efficient than poor-quality data from many sites.

^c^HCC: hepatocellular carcinoma.

^d^HBV: hepatitis B virus.

^e^HCV: hepatitis C virus.

^f^Surveillance for acute hepatitis cannot be used directly to quantify new infections. The reported number of cases of acute hepatitis needs to be adjusted for the large proportion of asymptomatic cases and underreporting.

**Table 4:** World Health Organization (WHO) surveillance case definitions for viral hepatitis. Case definitions are for the purpose of reporting and surveillance and may differ from criteria to be used for the management of patients.

Stage of infection	Criteria	Types of viral hepatitis			
		Hepatitis A	Hepatitis E	Hepatitis B	Hepatitis C
Acute hepatitis	Presumptive case: clinical criteria	Discrete onset of an acute illness with signs/symptoms of acute infectious illness (eg, fever, malaise, and fatigue) and liver damage (eg, anorexia, nausea, jaundice, dark urine, right upper quadrant tenderness, OR raised ALT^a^ levels more than 10 times the upper limit of normal of the laboratory)^b^	Discrete onset of an acute illness with signs/symptoms of acute infectious illness (eg, fever, malaise, and fatigue) and liver damage (eg, anorexia, nausea, jaundice, dark urine, right upper quadrant tenderness, OR raised ALT levels more than 10 times the upper limit of normal of the laboratory)	Discrete onset of an acute illness with signs/symptoms of acute infectious illness (eg, fever, malaise, and fatigue) and liver damage (eg, anorexia, nausea, jaundice, dark urine, right upper quadrant tenderness, OR raised ALT levels more than 10 times the upper limit of normal of the laboratory)	Discrete onset of an acute illness with signs/symptoms of acute infectious illness (eg, fever, malaise, and fatigue) and liver damage (eg, anorexia, nausea, jaundice, dark urine, right upper quadrant tenderness, OR raised ALT levels more than 10 times the upper limit of normal of the laboratory)
	Confirmed case: clinical criteria AND biomarker or epidemiological criteria	IgM^c^ anti-HAV^d^ +ve OR Epidemiological link with a confirmed case	IgM anti-HEV^e^ +ve OR Epidemiological link with a confirmed case	IgM anti-HBc^f^ +ve^g^	HCV^h^ RNA^i^ +ve and anti-HCV^j^ −ve OR Seroconversion to anti-HCV^k^ OR Anti-HCV +ve AND IgM anti-HBc −ve AND Anti-HAV IgM −ve AND Anti-HEV IgM −ve
Chronic infections (Only confirmed cases that all require clinical and biomarker criteria)	Clinical criteria	Not applicable	Rare event, no WHO standard case definition	Person *not* meeting the case definition for acute hepatitis^l^	Person *not* meeting the case definition for acute hepatitis^l^
	Biomarker criteria	Not applicable	Rare event, no WHO standard case definition	HBsAg +ve^m,n^	HCV RNA +ve OR HCV Ag +ve

^a^ALT: alanine aminotransferase.

^b^Ten times the upper limit of normal (400 IU/L) is the threshold used by the United States’ State and Territorial Epidemiologists (CSTE). Countries may also select lower (more sensitive) or higher (more specific) thresholds.

^c^Ig: immunoglobulin.

^d^anti-HAV: antibody against hepatitis A virus.

^e^anti-HEV: antibody against hepatitis E virus.

^f^anti-HBc: antibody against hepatitis B core antigen.

^g^Hepatitis test panels usually include HBsAg with anti-HBc IgM test (positive predictive value of anti-HBc IgM is higher if HBsAg is +ve). Specific test/threshold needed to exclude transient IgM during flares in chronic hepatitis B virus (HBV) infection.

^h^HCV: hepatitis C virus.

^i^RNA: ribonucleic acid.

^j^anti-HCV: antibody against hepatitis C virus.

^k^Among patients tested regularly at short time intervals, seroconversion to anti-HCV suggests a recent HCV infection. Seroconversions to anti-HCV should be followed by reflex RNA test (when available).

^l^Person tested in the context of the evaluation of a chronic liver disease, a check-up, or a survey.

^m^Most testing strategies would also test for total anti-HBc. The combination of total anti-HBc and HBsAg is more specific of chronic HBV infection than HBsAg alone.

^n^HBsAg: hepatitis B surface antigen.

#### Surveillance for Chronic Infections

The reference method for surveillance for chronic infections is regular biomarker surveys. When the opportunity to conduct population-based surveys is not available, *data mining* can also be undertaken to collate existing data on HBV or HCV infection [8]. Reporting cases of chronic infections present in health care facilities is not informative in terms of surveillance for chronic infections. This should be seen more as a way to monitor treatment in the context of a national database of patients with HBV or HCV infection (see the Patients Database section below) [8].

*Regular biomarker surveys* are the method of reference to estimate the prevalence of chronic infections in the general population. The viral hepatitis program can organize such biomarker surveys alone or in coordination with Demographic and Health Surveys, AIDS indicator surveys, or population HIV impact assessments that provide opportunities for coordination (depending on target populations, age groups, and sample sizes) [31]. Planning this integration ahead of time to include HIV, HBV, and HCV as part of the objectives of the survey may be better than testing stored specimens. Testing stored sera may raise methodological issues when specimens are not available from all study participants [40]. It can also raise ethical issues if participants identified with HBV or HCV cannot be linked to care [8]. The WHO prepared a template protocol to conduct biomarker surveys for viral hepatitis. This protocol will be available from the WHO upon request while in draft form and will be made available electronically on the Web when finalized. In addition to surveys conducted in the general population, surveys among specific groups (eg, PWID and men who have sex with men) can inform about risk behaviors and the prevalence of infection in these groups. This type of surveillance is often conducted in the context of HIV programs. Information from such surveys in specific populations may be used in combination with information from the general population to estimate the overall size of the infected population.

*Reporting of cases of chronic HBV and HCV infections* from health care facilities can be implemented in the context of a database of chronically infected patients (see the Patients Database section below) that documents the cascade of diagnosis, care, and treatment. Cases of chronic infection reported from health care facilities estimate the number of cases diagnosed. Reporting systems for these cases of chronic infections need to be completely separate from systems to report acute hepatitis. Reporting of cases of acute hepatitis is done for a different objective, which is to estimate incidence and identify risk factors for new infections. The practice of reporting cases of chronic infection together with cases of acute hepatitis may lead to a database that contains cases of acute hepatitis diluted in a larger number of chronic infections. This complicates interpretation of the data on acute hepatitis. Acute hepatitis data are particularly useful because they reflect recent infections. If they are merged with data on chronic infections for which the date of infection is unknown, they lose specificity and usefulness.

#### Surveillance for Sequelae (Including Mortality)

Viral hepatitis–associated mortality is spread across various causes of death in vital registration systems. These include acute hepatitis (that accounts for a small proportion of deaths), cirrhosis, and hepatocellular carcinoma (for which the link with HBV and HCV infection is not documented in the death certificates) [41]. To quantify deaths from the sequelae of HBV and HCV infections, the WHO proposes to start from the mortality envelope from sequelae (the deaths from cirrhosis and hepatocellular carcinoma from vital registration data) and to correct it on the basis of the fraction of these sequelae that are attributable to HBV and HCV infection. This may be done using national data on the prevalence of HBV and HCV infection among patients with cirrhosis and hepatocellular carcinoma. Such estimates may be obtained from published studies, unpublished data, or regional estimates [18,42].

### Patient Database for Testing and Treatment

At an early stage of a program, estimates describing the cascade of testing and treatment may be obtained from ad hoc mechanisms (eg, surveys and data on sales of medicines). However, the best approach to monitor and evaluate a national program for testing and treatment of HBV and HCV infection is to establish a national database of persons with chronic infections.

If feasible, combining case reporting and patient monitoring systems can create a national database of patients with chronic HBV and HCV infection. Health care providers can use such databases to manage data on personal characteristics, diagnosis, treatment initiation or deferral, monitoring, and viral suppression/cure. When a person is diagnosed (ie, newly identified cases of chronic infection), his/her record is added to the database. The record is censored when the person is cured or dies. The system may be made of standardized patient cards, paper registers, or an electronic data entry system. If data collection is paper-based, information then needs to be entered on a computer. Unique identifiers are necessary to identify and remove duplicate reports (ie, deduplication) and to protect confidentiality of patients. They can also be used to follow individuals along the cascade of services over the medium term and as they move between facilities. Automated data analysis can then aggregate individual data and calculate core indicators on treatment coverage/initiation (C.7.a/C.7.b) and viral suppression/cure (C.8.a/C.8.b) using a cohort approach. From 2017, the WHO will be assisting countries through the preparation of a template patient card, database metadata, and analyses plans that will allow setting up electronic patient registries. However, ideally, such a database should be compatible with other existing health information systems, such as the one used for HIV.

### Coinfections

About 2.3 million people living with HIV are coinfected with HCV [43] and 2.6 million with HBV [44,45]. HBV-HIV coinfection became easier to handle as the first-line recommended antiretroviral regimens now include tenofovir, which would also effectively treat HBV infection [46]. With respect to HCV-HIV coinfection, testing of HIV-infected patients for HCV infection is necessary as coinfection requires specific management and treatment [13]. HCV treatment may be administered before HIV treatment if the patient is not immunosuppressed. If the patient is immunosuppressed, HIV treatment needs to be started first, and the HCV treatment regimen will need to be adapted to avoid drug interactions [13].

The consolidated strategic information guidelines for HIV included two indicators for coinfection, LINK 27 for HBV and LINK 28 for HCV [7]. LINK 27 and LINK 28 reflect the proportion of patients in care for HIV that have been screened for HBV and HCV, respectively. In 2016, in view of the new “treat all” HIV treatment guidelines, the WHO proposed to amend the LINK 27/28 indicators so that the numerator would be the number of persons newly placed on anti-retroviral treatment screened for hepatitis B/C during the reporting period and the denominator would be the number of persons newly placed on ART. For HCV infection, testing for HCV infection would mean implementing the full testing strategy that includes HCV nucleic acid test testing if the patient is anti-HCV positive. This modification would best reflect the need to look for hepatitis before starting treatment. It will help ensure that HBV-infected patients are placed on tenofovir-based therapy and that HCV-infected people are being considered for specific treatment.

### Conclusions

The many commonalities between HIV, HBV, and HCV in terms of the diseases that they cause and response required justify implementation of the programs with coordination, strategic linkages, or integration, according to the respective magnitudes of the HIV and viral hepatitis epidemics. These articulations should be considered at the strategic, policy, technical, and implementation levels. Alignment of the collection, analysis, and use of strategic information are critical areas of this coordinated implementation. The WHO monitoring and evaluation frameworks for HIV, HBV, and HCV have been constructed using a similar logic to facilitate alignment. However, the differences between HIV, HBV, and HCV call for collaborations with other areas of work to ensure a comprehensive approach. These include communicable disease surveillance for acute hepatitis surveillance, immunization, and hepatology centers for sequelae surveillance. The focus of the SDGs on a single health goal and the similarities in the targets for HIV, HBV, HCV, and tuberculosis mean that the overarching priority would be to integrate viral hepatitis strategic information and HIV strategic information within the broader existing health information systems and thus contribute toward strengthening the health system. With this approach, a coordinated incremental investment in the data system will provide the evidence base needed to guide elimination of viral hepatitis.

## Appendix

Multimedia Appendix 1 Monitoring and evaluation framework.

## Abbreviations

AIDS: acquired immune deficiency syndrome
ALT: alanine aminotransferase
anti-HAV: antibody against hepatitis A virus
anti-HBc: antibody against hepatitis B core antigen
anti-HCV: antibody against hepatitis C virus
anti-HEV: antibody against hepatitis E virus
GHSS: Global Health Sector Strategy
HBIg: Hepatitis B immune globulin
HBsAg: Hepatitis B surface antigen
HBV: hepatitis B virus
HCC: hepatocellular carcinoma
HCV: hepatitis C virus
Ig: immunoglobulin
PWID: people who inject drugs
RNA: ribonucleic acid
SARA: Service Availability and Readiness Assessment
SDGs: Sustainable Development Goals
STIs: sexually transmitted infections
UNICEF: United Nations International Children’s Emergency Fund
WHO: World Health Organization

## References

[1] (2017). World Health Organization.

[2] GBD 2013 Mortality and Causes of Death Collaborators (2015). Global, regional, and national age-sex specific all-cause and cause-specific mortality for 240 causes of death, 1990-2013: a systematic analysis for the Global Burden of Disease Study 2013. Lancet.

[3] (2016). World Health Organization.

[4] (2016). World Health Organization.

[5] World Health Organization.

[6] No authors listed (2009). Hepatitis B vaccines. Wkly Epidemiol Rec.

[7] (2015). World Health Organization.

[8] (2016). World Health Organization.

[9] (2016). World Health Organization.

[10] Fenton KA, Aquino GA, Dean HD (2014). Program collaboration and service integration in the prevention and control of HIV infection, viral hepatitis, STDs, and tuberculosis in the U.S.: lessons learned from the field. Public Health Rep.

[11] Okulicz F, Lambotte O (2011). Epidemiology and clinical characteristics of elite controllers. Curr Opin HIV AIDS.

[12] Edmunds WJ, Medley GF, Nokes DJ, Hall AJ, Whittle HC (1993). The influence of age on the development of the hepatitis B carrier state. Proc Biol Sci.

[13] World Health Organization (2016). Guidelines for the screening, care and treatment of persons with chronic hepatitis C infection.

[14] Stover J, Johnson P, Zaba B, Zwahlen M, Dabis F, Ekpini RE (2008). The Spectrum projection package: improvements in estimating mortality, ART needs, PMTCT impact and uncertainty bounds. Sex Transm Infect.

[15] Chen JD, Yang HI, Iloeje UH, You SL, Lu SN, Wang LY, Su J, Sun CA, Liaw YF, Chen CJ, Risk Evaluation of Viral Load Elevation and Associated Liver Disease/Cancer in HBV (REVEAL-HBV) Study Group (2010). Carriers of inactive hepatitis B virus are still at risk for hepatocellular carcinoma and liver-related death. Gastroenterology.

[16] Chu C, Liaw Y (2009). Incidence and risk factors of progression to cirrhosis in inactive carriers of hepatitis B virus. Am J Gastroenterol.

[17] Thein HH, Yi Q, Dore GJ, Krahn MD (2008). Estimation of stage-specific fibrosis progression rates in chronic hepatitis C virus infection: a meta-analysis and meta-regression. Hepatology.

[18] Perz JF, Armstrong GL, Farrington LA, Hutin YJ, Bell BP (2006). The contributions of hepatitis B virus and hepatitis C virus infections to cirrhosis and primary liver cancer worldwide. J Hepatol.

[19] Ramos-Casals M, Zignego AL, Ferri C, Brito-Zerón P, Retamozo S, Casato M, Lamprecht P, Mangia A, Saadoun D, Tzioufas AG, Younossi ZM, Cacoub P, International Study Group of Extrahepatic Manifestations related to HCV (ISG-EHCV) (2017). Evidence-based recommendations on the management of extrahepatic manifestations of chronic hepatitis C virus infection. J Hepatol.

[20] Pépin J, Abou Chakra CN, Pépin E, Nault V, Valiquette L (2014). Evolution of the global burden of viral infections from unsafe medical injections, 2000-2010. PLoS One.

[21] Vun MC, Galang RR, Fujita M, Killam W, Gokhale R, Pitman J, Selenic D, Mam S, Mom C, Fontenille D, Rouet F, Vonthanak S, Roka Cluster Investigation Team (2016). Cluster of HIV infections attributed to unsafe injection practices--Cambodia, December 1, 2014-February 28, 2015. MMWR Morb Mortal Wkly Rep.

[22] Yeung CY, Lee HC, Chan WT, Jiang CB, Chang SW, Chuang CK (2014). Vertical transmission of hepatitis C virus: current knowledge and perspectives. World J Hepatol.

[23] (2015). World Health Organization.

[24] (2015). Sustainabledevelopment.un.

[25] (2016). World Health Organization.

[26] Nayagam S, Thursz M, Sicuri E, Conteh L, Wiktor S, Low-Beer D, Hallett TB (2016). Requirements for global elimination of hepatitis B: a modelling study. Lancet Infect Dis.

[27] (2012). World Health Organization, United Nations Office on Drugs and Crime, Joint United Nations Programme on HIV/AIDS.

[28] (2008). World Health Organization.

[29] Mast EE, Alter MJ, Margolis HS (1999). Strategies to prevent and control hepatitis B and C virus infections: a global perspective. Vaccine.

[30] (2016). World Health Organization.

[31] UNAIDS/WHO Working Group on Global HIV/AIDS and STI Surveillance 2015 (2015). Unaids.

[32] World Health Organization.

[33] (2015). World Health Organization.

[34] (2017). World Health Organization and Joint United Nations Programme on HIV/AIDS.

[35] Werner BG, Grady GF (1982). Accidental hepatitis-B-surface-antigen-positive inoculations. Use of e antigen to estimate infectivity. Ann Intern Med.

[36] Jagger J, Puro V, De Carli G (2002). Occupational transmission of hepatitis C virus. J Am Med Assoc.

[37] Baggaley RF, Boily MC, White RG, Alary M (2006). Risk of HIV-1 transmission for parenteral exposure and blood transfusion: a systematic review and meta-analysis. AIDS.

[38] Van Handel MM, Rose CE, Hallisey EJ, Kolling JL, Zibbell JE, Lewis B, Bohm MK, Jones CM, Flanagan BE, Siddiqi AE, Iqbal K, Dent AL, Mermin JH, McCray E, Ward JW, Brooks JT (2016). County-level vulnerability assessment for rapid dissemination of HIV or HCV infections among persons who inject drugs, United States. J Acquir Immune Defic Syndr.

[39] Harris AM, Iqbal K, Schillie S, Britton J, Kainer MA, Tressler S, Vellozzi C (2016). Increases in acute hepatitis B virus infections - Kentucky, Tennessee, and West Virginia, 2006-2013. MMWR Morb Mortal Wkly Rep.

[40] Ly KN, Kim AA, Umuro M, Drobenuic J, Williamson JM, Montgomery JM, Fields BS, Teshale EH (2016). Prevalence of hepatitis B virus infection in Kenya, 2007. Am J Trop Med Hyg.

[41] Mahajan R, Xing J, Liu SJ, Ly KN, Moorman AC, Rupp L, Xu F, Holmberg SD, Chronic Hepatitis Cohort Study (CHeCS) Investigators (2014). Mortality among persons in care with hepatitis C virus infection: the Chronic Hepatitis Cohort Study (CHeCS), 2006-2010. Clin Infect Dis.

[42] de Martel C, Maucort-Boulch D, Plummer M, Franceschi S (2015). World-wide relative contribution of hepatitis B and C viruses in hepatocellular carcinoma. Hepatology.

[43] Platt L, Easterbrook P, Gower E, McDonald B, Sabin K, McGowan C, Yanny I, Razavi H, Vickerman P (2016). Prevalence and burden of HCV co-infection in people living with HIV: a global systematic review and meta-analysis. Lancet Infect Dis.

[44] Easterbrook P, Platt L, Gower E, Razavi H, Sabin K, Vickerman P (2015). Global systematic review and meta-analysis of the seroprevalence of HBV and HCV infection in HIV-infected persons. TUPEB 254 abstract.

[45] Kourtis AP, Bulterys M, Hu DJ, Jamieson DJ (2012). HIV–HBV coinfection — a global challenge. N Engl J Med.

[46] (2016). World Health Organization.

