# Supplementary material for: Viral Hepatitis Strategic Information to Achieve Elimination by 2030: Key Elements for HIV Program Managers
Source: JMIR Public Health Surveill. 2017 Dec 15;3(4):e91. doi: 10.2196/publichealth.7370 (PMC5747598; doi:10.2196/publichealth.7370)
Supplement: Multimedia Appendix 1 [file publichealth_v3i4e91_app1.pdf]

Monitoring and evaluation framework: minimum set of 10 core indicators to monitor and evaluate the health sector response to viral hepatitis B and C along the result chain in countries.

	Epidemics	System	Outputs and outcomes						Elimination	
			Prevention			Cascade of care				
	C.1 Prevalence of chronic infections	C.2 Capacity for testing	C.3 Coverage of PMTCT[a] and vaccination	C.4-5 Prevention of transmission of blood-borne pathogens		C.6 Proportion of patients diagnosed	C.7 Treatment	C.8 Outcome of treatment among those treated	C.9 Incidence	C.10 Mortality from HCC[b] and cirrhosis
**Virus**										
HBV	C.1.a HBV[c] (HBsAg)[d]	Number of health care facilities able to test for HBV infection per 100,000 population	C.3.a Timely HBV vaccine birth dose and C.3.b 3rd dose HBV vaccine	C.4 Harm reduction (needle and syringe distribution)	C. 5 Injection safety (facility-level injection safety)	C.6.a HBV	C.7.a Coverage	C.8.a Viral suppression	C.9.a Cumulated incidence of HBV infection in children 5 years of age	C.10.a HBV deaths
HCV[e]	C.1.b HCV (HCV RNA[f]/CoreAg)	Number of health care facilities able to test for HCV infection per 100,000 population	Not applicable	C.4 Harm reduction (needle and syringe distribution)	C. 5 Injection safety (facility-level injection safety)	C.6.b HCV	C.7.b Initiation	C.8.b Cure	C.9.b Incidence of HCV infection	C.10.b HCV deaths
**Practical implementation of the M&E framework**										
Data system required	Biomarker surveys	Health care facility surveys	EPI[g] routine data	Program data from harm reduction	Health care facility surveys	Patients database	Patients database	Patients database	Biomarker surveys	Vital registration, cancer registries, and hepatology centers data
Opportunities for coordination	HIV surveys and immunization surveys	SARA[h]	EPI	Harm reduction and HIV	SARA	HIV	HIV	HIV	HIV surveys and immunization surveys	—

[a]PMTCT: prevention of mother-to-child transmission.
[b]HCC: hepatocellular carcinoma.
[c]HBV: hepatitis B virus.
[d]HBsAg: hepatitis B surface antigen.
[e]HCV: hepatitis C virus.
[f]RNA: ribonucleic acid.
[g]EPI: Expanded Program on Immunization.
[h]SARA: Service Availability and Readiness Assessment.
